# Prevalence and Associations of Depression among Saudi College Nursing Students: A Cross-Sectional Study

**DOI:** 10.3390/healthcare12131316

**Published:** 2024-07-01

**Authors:** Salman Alreshidi, Ahmad Rayani, Ahmad Aboshaiqah, Ahmed Aljaloud, Sanaa Ghulman, Abdalhadi Alotibi

**Affiliations:** 1 Community and Psychiatric Mental Health Nursing Department, College of Nursing, King Saud University, Riyadh City P.O. Box 12372, Saudi Arabia; arayani@ksu.edu.sa; 2 Department of Nursing Administration and Education, College of Nursing, King Saud University, Riyadh City P.O. Box 12372, Saudi Arabia; aaboshaiqah@ksu.edu.sa; 3 King Saud University Medical City, Riyadh City P.O. Box 12372, Saudi Arabia; aaljaloud@ksu.edu.sa (A.A.); sghulman@ksu.edu.sa (S.G.); aabdalhadi@ksu.edu.sa (A.A.)

**Keywords:** Center for Epidemiological Studies Depression, Saudi Arabia, risk factors, college nursing students, prevalence, depression

## Abstract

Depression affects approximately 350 million individuals globally and is the leading cause of disability. Depression among nursing students is an ongoing issue, yet little is known about the relationship between depression and its risk factors among nursing students in Saudi Arabia. The purpose of this study was to determine the prevalence of depression among a cohort of Saudi nursing students and to explore the risk factors associated with depression in this group. A cross-sectional design was employed for this study, which was conducted by the nursing faculty at King Saud University in Riyadh, Saudi Arabia. The researcher sampled 330 nursing students, with 252 students (a 76.3% response rate) randomly selected by the academic advising unit in the nursing college between January and February 2023. The participants completed an online survey based on the Arabic version of the Center for Epidemiologic Studies Depression Scale. The data were analyzed using univariate analysis and backward multi-variable logistic regression. The findings revealed that 65.1% of the participants scored 16 or above on the depression scale, indicating high levels of depressive symptoms. Univariate analysis identified several significant risk factors for depression, including sex (OR, 0.29; 95% CI, 0.16–0.51; *p* < 0.001), academic pressure (OR, 5.87; 95% CI, 2.04–16.84; *p* < 0.001), interpersonal relationships (evaluated by balance and harmony in thoughts, emotions, behavior, and interactions with others; categorized as good/fair or poor), and the relationship with the father, which were strongly associated with the presence of depression symptoms. Backward multi-variable logistic regression analysis further revealed that being male, experiencing reduced academic pressure, having stronger father–son relationships, and maintaining positive interpersonal relationships were significantly associated with decreased levels of depression (ORs ranged from 0.25 to 3.94). These findings highlight the need for family and school-based prevention interventions to help nursing students in college avoid depression.

## 1. Introduction

Depression is regarded as a multi-problematic neurological condition that impairs an individual’s ability to function on a social, interpersonal, and professional level. Studies have found a link between depression and suicidal behavior in college students [1,2,3]. Thus, there is a pressing need to develop methods of identifying college students who are at risk of depression [4,5]. According to data published by the American College Health Association in 2019, 30.7% of college students contemplated suicide and were experiencing depression [6]. Approximately 60% of individuals who commit suicide are suffering from severe depression at the time at which they choose to end their lives, causing considerable pain for their loved ones and those in their immediate environment [7]. Thus, the prevalence and associations of depression among college students globally have attracted significant attention in recent years [8,9,10].

The findings of prior studies indicate that the prevalence of depression among college students differs significantly between settings, with educational tools and cultural contexts being pertinent predictors [3]. Earlier research found that depression rates vary between 3% and 80.6% among Chinese college students and between 21.2% and 27.8% among young adults in the United States and Indonesia [11,12,13]. Herman and Archambeau [14] concluded that the prevalence of depression observed in the three most prominent U.S. ethnic racial groups (African Americans, European Americans, and Hispanics) was not consistent. They found that African Americans and Hispanic Americans reported more symptoms of depression than their European American counterparts. However, other studies found no significant variations or differences depending on how depression was defined [14].

Nurses in the medical field must deal with a great deal of academic and psychological pressure. As such, they are more susceptible to depression than those working in alternative industries [3,15,16]. Given the professional stress and uncertainty of the profession, nursing students will likely face psychological pressure as they have regular and direct interactions with patients, impacting their behavior and attitudes. Clinical placement for undergraduate students begins in their second year of study and gradually increases to 16 weeks in their final year, followed by 52 weeks of internship site training [17]. Depression may impair nurses’ performance and negatively affect their interactions with patients [18,19]. Hospitals and society have experienced increased depression among nurses in recent years [20,21,22].

Studies involving 2111 undergraduate nursing students studying at various institutions in the United States and 386 undergraduate nursing students in Turkey found that 16% and 17.4%, respectively, of those surveyed reported experiencing moderate major depressive disorder symptoms [23,24]. Nursing degrees and courses of study involve a heavy workload, stressful clinical practice, and high-pressure academic settings. In addition to their challenging study requirements, student nurses must navigate various personal and social factors. These factors include dealing with interpersonal issues, maintaining physical activity, managing academic performance, addressing the impacts of being an only child, their residential status, genuine interest in the nursing field, job prospects, and various aspects of their family background. The latter includes the educational levels of their parents, the quality of their relationships with parents, and their independence in decision-making. Collectively, these stressors can significantly heighten nursing students’ risk of experiencing depression and suicidal thoughts [25,26].

Goetz attributed the fact that students majoring in nursing are at a higher risk of experiencing suicidal thoughts than individuals studying alternative majors to the high-pressure academic curriculum nursing students encounter [27]. One study focusing on nursing students in Greece reported that 10.6% of the participants had experienced suicidal ideation at some point in their lives. Additionally, the study revealed that 1.7% of female participants disclosed they would consider dying by suicide if given the opportunity [28]. Therefore, it is crucial to investigate the symptoms of depression in nursing students to develop appropriate clinical interventions.

However, only a few studies have examined the prevalence and associations of depression among Saudi nursing students in Riyadh province [15,29]. Understanding the prevalence of depression is vital, given that the onset of most lifetime depression typically occurs during college years [30,31]. Additionally, gaining insights into the mental health of university nursing students could have significant implications for campus health services and the development of mental health policies for this vulnerable group. As future healthcare professionals, it is essential to thoroughly understand nursing students so they can be better prepared to assist others upon graduation. The specific objectives of this study were to (i) estimate the prevalence rates of depression among a cohort of Saudi nursing students, (ii) compare their average scores to normative values in Saudi Arabia and internationally among college students, and (iii) identify educational, socio-demographic, and family-related predictors associated with the prevalence of depression.

## 2. Materials and Methods

### 2.1. Participants

Using a cross-sectional design, the researcher randomly sampled nursing students from the College of Nursing, King Saud University, in Riyadh City, Saudi Arabia. The College of Nursing at King Saud University has a total of 1326 nursing students [32]. Using Google Forms, a link comprising the consent form, demographic items, and questionnaire was sent to 330 nursing students via email, randomly selected by the academic advising unit in the nursing college between January and February 2023, with 252 responses (representing a 76.3% response rate). All the fields in the Google Forms link were assigned to be mandatory to prevent any missing data. Students were given descriptions of the study’s goals. They were informed they could stop participating in the study at any time. Participants were reassured that their responses would remain anonymous and private. The STROBE checklist guided the report of this study for an observational study.

Selection criteria included nursing students who (a) consented to participate in this study and (b) were currently enrolled as full-time Bachelor of Nursing Degree students. Exclusion criteria included (a) first-year students and (b) students who had a history of psychiatric disease.

When multiple regressions were considered, it was determined that a sample size of 141 was needed based on the previously described effect size of 0.39 [33]. The effect sizes were categorized as small (0.15), medium (0.39), and large (0.59). This study’s model incorporated 16 predictors and a significance level of 0.05 to achieve the required minimum level of statistical power of 0.8.

### 2.2. Instruments

The participants were provided with the Arabic version of the Center for Epidemiologic Studies Depression Scale (CES-D) [34,35]. This tool consisted of 20 items that the respondents responded to using a four-point Likert scale that ranged from “rarely or none of the time” (value = 0) to “most or all of the time” (value = 3). The respondents were asked to reflect on any feelings they had experienced regarding each item over the past week. The sum of the response scores ranged from 0 to 60. In the current study, the CES-D scale demonstrated good internal consistency and reliability according to Spearman–Brown and Cronbach’s alpha coefficients of 0.851 and 0.844, respectively. The conventional cutoff (CES-D N = 16), which reflects a participant at risk for clinical depression, was used to determine whether a participant reported clinically relevant levels of depression. More specifically, the CES-D cut points are: “0 to 16 points: no to minimal depression”, “16 to 23 points: moderate depression”, and “24 to 60 points: severe depression”.

Additionally, this study gathered data on the participants’ characteristics, including gender, whether they were a single child, their prior residence (urban or rural), grades, perceived academic pressure (have greater/medium/less academic pressure), perceived interest in nursing major (have greater/medium/less interest in nursing major), perceived future employment prospects (have good/fair or poor future employment prospective in nursing major), perceived interpersonal relationships (by finding balance and harmony in one’s thinking, emotions, behavior, and interactions with others; having good/fair or poor interpersonal relationship with other), frequency of exercise, and family background (parents’ educational levels, the participants’ relationships with their parents, and their ability to make decisions without parental interference). All factors were divided into two, three, or four groups (Table 1).

### 2.3. Statistical Evaluation

Chi-square analysis and a *t*-test were employed to assess the association between symptoms of depression and categorical predictors and continuous predictors, respectively. To calculate the relationship between statistically significant predictors (*p* < 0.05) in the chi-square/*t*-test analysis and depression, a multi-variable logistic regression model using the backward approach (*p*_out_ = 0.10) was utilized. OR and related 95% CI were estimated to evaluate the association between the previously described risk factors and the presence of symptoms of depression. Using SPSS 28.0 for Windows (SPSS Inc., Chicago, IL, USA) to evaluate the data, a *p*-value of 0.05 or lower was deemed significant.

## 3. Results

The findings revealed that the overall prevalence of depression among the study population of nursing students was 65.1% (95%CI: 20.3%–23.4%). In males, the prevalence was 52.9% (95%CI: 17.1%–21.1%), lower than the 79.3% (95%CI: 22.8%–27.4%) among the female members of the population. The mean score of CES-D was 3.1 times that of the responses of the individuals in the depressed group (CES-D of 16 or higher) (28.7 ± 9.89 vs. 9.2 ± 3.85, *p* < 0.001). In terms of the respondents allocated to the depressed group (CES-D ≥ 16), 56.1% were females, while 52.4% reported having greater academic pressure. In the group of nondepressed students, 72.7% were males, while 61.4% reported having medium academic pressure. Most participants, 98%, were not a single child in their family, and 92.9% were residents of urban cities (Table 1). In this study, 34.9% of participants experienced no-to-minimal depression. On the contrary, 23.8% and 41.3% of the study population reported moderate and severe depression, respectively (Table 2).

Table 3 shows that the univariate regression analysis revealed significant differences in the risk of depression symptoms among nursing students. Male nursing students had a 71% lower OR for being at risk of depressive symptoms compared to female students (OR, 0.29; 95% CI, 0.16–0.51; *p* < 0.001). Additionally, nursing students experiencing higher academic pressure were approximately six times more likely to exhibit symptoms of depression compared to those with lower academic pressure (OR, 5.87; 95% CI, 2.04–16.84; *p* < 0.001). Students with good interpersonal relationships were 68% less likely to be at elevated risk of depressive symptoms (OR, 0.32; 95% CI, 0.18–0.56; *p* < 0.001). Furthermore, those with a strong relationship with their father were 80% less likely to experience depressive symptoms compared to those with fair or poor relationships with their father (OR, 0.20; 95% CI, 0.09–0.44; *p* < 0.001).

Following the backward method, four significant predictors were included in the multi-variable logistic model, with a *p*-value cutoff of 0.10. Compared to pre-adjustment levels, the associations between these predictors and symptoms of depression were attenuated, resulting in ORs ranging from 0.25 to 3.94. Participants experiencing greater academic pressure were 394% more likely to suffer from depression compared to those with less academic pressure (OR, 3.94; 95% CI, 1.23–12.54; *p* = 0.020). Moreover, having a strong relationship with one’s father reduced the likelihood of depression by 75% compared to having fair or poor relationships with fathers (OR, 0.25; 95% CI, 0.11–0.58; *p* < 0.001). Similarly, having good interpersonal relationships lowered the risk of depression (OR, 0.36; 95% CI, 0.20–0.67; *p* < 0.001), and being male reduced the likelihood of experiencing depressive symptoms (OR, 0.42; 95% CI, 0.23–0.87; *p* < 0.001). However, the association between moderate academic pressure and depression was not statistically significant according to the multi-variable logistic regression model (Table 3).

The results of the linear regression analysis, as shown in Supplementary Table S1, revealed that four predictors accounted for 34.7% of the variation in depression score that was observed: academic pressure (β = 0.212, *p* < 0.001), interpersonal relationships (β = −0.190, *p* < 0.001), relationship with father (β = −0.188, *p* = 0.003), and relationship with mother (β = −0.238, *p* < 0.001). Notably, academic pressure positively impacted depression, but interpersonal relationships and relationships with father and mother negatively impacted depression.

## 4. Discussion

According to the study outcomes, 65.1% of participants had depression. The likelihood of nursing students experiencing symptoms of depression was significantly enhanced by being female, having poor interpersonal relationships, and experiencing academic pressure. Being on good terms with parents and making independent decisions were found to be negatively associated with depression symptoms. Other predictors, including exercise frequency and parents’ educational attainment, were also weakly associated with depressive symptoms. These findings suggest that efforts aimed at preventing and treating depression in Saudi nursing students should concentrate on assisting students in managing academic pressure, enhancing their future career prospects, establishing positive family relationships, and engaging their parents in their lives.

The results of the current study were similar to those of studies performed in other nations, such as Turkey (50%), Greece (44%), and Thailand (47%) [24,28,36]. They are also comparable to those of baccalaureate nursing students from Saudi Arabia in Riyadh province (44.2%, and 43.3%) and Medina province (58.7%) [15,29,37], which showed that 65.1% of the respondents exhibited symptoms of depression (16 or above on the CES-D scale). However, another study indicated that the depression rate among medical students in Africa was lower (40.9%) [38]. As previously described, most Saudi nurses are women, who play a crucial role in the country’s healthcare system. The findings of the current study revealed that 56.1% of the female respondents were assessed as “depressed” compared to 43.9% of males. The cause of the high occurrence of depression among nurses is unknown compared to non-nursing students; however, it is understood that there are many contributing factors, including psychological issues resulting from the high demands of their work, caring for the sick, and frequent contact with severely ill patients [39].

Slight differences across the different studies can be attributed to the use of varying depression scales; for instance, the Beck Depression Inventory (BDI), CES-D, Hamilton, and Zung [40]. These scales do not cover the same common depressive symptom factors as those described by Shafer [40]. Additionally, the period in which the study was performed may have led to variations in the prevalence of depression noted across the different studies. Some studies have found that seasonal weather patterns may affect the release of neuromodulators, including serotonin [41,42]. Sociodemographic settings may also influence slight differences in depression severity [14]; however, these do not alter this study’s interpretive findings.

According to the findings of the current study, there was a high association between academic pressure and depression among nursing students. This finding aligns with prior research involving samples from students in Saudi and Arabic settings [15,43,44,45]. Using Seidman’s guidance to perform in-depth qualitative interviews, researchers in another study on depressed American college students discovered that academic and professional issues were the main drivers of stress [10]. According to the aforementioned studies, academic pressure is a “typical” and universal problem that Eastern and Western college students, especially nursing students, frequently encounter. Nursing students typically have a higher academic load, study in a more competitive environment, and have fewer job opportunities after graduation in the fast-paced contemporary world. Consequently, nursing students are under more pressure to perform well in class to meet parental expectations and equip themselves with the necessary skills for the future. These factors may negatively affect their stress levels [15,46,47]. This can increase nurses’ stress regarding their academic pressure, leading to the emergence of depressive symptoms. Therefore, stress management may be a crucial skill that nursing students should be taught to master. Efficient interventions and education can help address these issues. For example, the creation of peer discussion groups can assist students in processing conflicts and help them reflect on the fact that their situations are not exceptional, thereby relieving their stress [48].

Numerous studies have demonstrated the significant impact of family harmony and support on depression and suicidal ideation within a population of college students [49,50,51]. One recent systemic review indicated that depression in fathers has comparable effects on fathers’ parenting and relationships with their children from the age of two months to twenty-one years [52]. In addition, greater levels of parent-adolescent conflict and lower levels of family cohesiveness and engagement are linked with higher rates of depression and suicidal thoughts. Our findings strongly support the outcomes of previous research, which has demonstrated that a supportive and stable family environment (such as a positive relationship between children and their parents or a child’s ability to make decisions without parental interference) may help teenagers and young adults develop better personal attributes. The natural process helps alleviate melancholia. Consequently, some researchers have suggested family-based therapy as a required intervention [53,54].

A multi-variable model performed among Korean and Chinese nursing students found no significant association between interest in their major and depression [55]. Additionally, the present study did not reveal a significant link between exercise frequency and depression. This finding was contrary to previous studies [56,57]. These disparities can be partially attributed to differences in the covariates that were considered in the data analysis, the lack of an objective framework that can adequately measure physical activity, the lack of physical activity among the participants compared to those living in developed nations, and the ages of the study participants, which were restricted to those aged between 17 and 22. In the current study, parental education level was not substantially related to depression. The relationship between family sociodemographic traits, particularly parents’ educational attainment, and depression in medical students requires further research.

The cross-sectional methodology of this study is a possible limitation because it renders it challenging to conclude the causes of depression. The causal association between the prevalence of depression and the risk factors examined in the current investigation should be established in future follow-up studies. Furthermore, the findings of this study must be evaluated carefully because all measurements were self-reported. However, the study participants were guaranteed confidentiality through anonymity and informed that their responses would not result in any consequences. This may have reduced the likelihood that students would hide their true feelings. Moreover, the study’s sample was limited to a single university, potentially limiting its generalizability. Finally, the questionnaire did not account for potential confounders such as social support, especially family support, self-efficacy, smoking status, diet quality, and sleep quality details, which could affect depression symptoms [58].

### Implications for Practice

Depression among university nursing students can have implications for their health, brain development, quality of life, behavior, and academic performance. The repercussions extend beyond the individual, affecting their families, educational institutions, and potentially the well-being of others they interact with. Consequently, depression can severely impair a student’s quality of life, potentially leading to suicidal outcomes. While this study provides an estimate of the prevalence of depression in this group, it is crucial to focus on students exhibiting symptoms of depression. In addition, government and educational authorities may consider implementing measures such as creating awareness programs, providing early intervention for at-risk students, and ensuring access to psychiatric support and services. Additionally, universities could consider appointing psychologists or psychiatric nurses within each department to enhance mental health support for nursing students. Ultimately, the findings from this study, including the predictors of depression, could inform clinical psychiatric nursing practices aimed at addressing mental health issues among university students.

## 5. Conclusions

The current study makes a significant contribution to the existing literature; however, further objective studies are required to precisely identify the predictors that can cause symptoms of depression in nursing students. The risk factors for depression among nursing students in Saudi Arabia highlighted in this study are significant. The findings of this research also have significant repercussions for the healthcare industry that professionals would take into consideration when seeking to implement effective strategies at the familial, academic, and societal levels to address depression and foster Saudi Arabian nursing students to become competent nurses with a positive mental state.

## Figures and Tables

**Table 1:** The association between education, socio-demographic characteristics and family-related predictors and depression in a population of Saudi nursing college students (N = 252).

	n (%)	Non-Depressed (CES-D < 16) (n = 88)	Depressed (CES-D ≥ 16) (n = 164)	*p*
CES-D score ^a^		9.2 ± 3.85	28.7 ± 9.89	<0.001
**Personal information**				
Sex ^b^				<0.001
Male	136 (54)	64 (72.7)	72 (43.9)	
Female	116 (46)	24 (27.3)	92 (56.1)	
Single child ^b^				0.810
Yes	5 (2)	2 (2.3)	3 (1.8)	
No	247 (98)	86 (97.7)	161 (98.2)	
Place of residence before college ^b^				0.509
Rural	18 (7.1)	5 (5.7)	13 (7.9)	
Urban	234 (92.9)	83 (94.3)	151 (92.1)	
Grades b				0.807
Better	225 (89.3)	78 (88.6)	147 (89.6)	
Medium or Less	27 (10.7)	10 (11.4)	17 (10.4)	
Academic pressure ^b^				<0.001
Greater	109 (43.3)	23 (26.1)	86 (52.4)	
Medium	125 (49.6)	54 (61.4)	71 (43.3)	
Less	18 (7.1)	11 (12.5)	7 (4.3)	
Interest in their major ^b^				0.657
Greater	114 (45.2)	41 (46.6)	73 (44.5)	
Medium	114 (45.2)	37 (42)	77 (47)	
Less	24 (9.5)	10 (11.4)	14 (8.5)	
Perspective on future career prospects b				0.422
Good	178 (70.6)	66 (75)	112 (68.3)	
Fair or Poor	74 (29.4)	22 (25)	52 (31.7)	
Interpersonal relationships ^b^				<0.001
Good	140 (55.6)	64 (72.7)	76 (46.3)	
Fair or Poor	112 (44.4)	24 (27.3)	88 (53.7)	
Exercise frequency ^b^				0.002
No	117 (46.4)	27 (30.7)	90 (54.9)	
1–2 days/week	69 (27.4)	30 (34.1)	39 (23.8)	
3–5 days/week	52 (20.6)	23 (26.1)	29 (17.7)	
>5 days/week	14 (5.6)	8 (9.1)	6 (3.7)	
**Family background**				
Educational level of father ^b^				0.840
<High school	81 (32.1)	29 (33)	52 (31.7)	
≥High school	171 (67.9)	59 (67)	112 (68.3)	
Educational level of mother ^b^				0.811
<High school	120 (47.6)	41 (46.6)	79 (48.2)	
≥High school	132 (52.4)	47 (53.4)	85 (51.8)	
Relationship with father ^b^				<0.001
Good	185 (73.4)	79 (89.8)	106 (64.4)	
Fair or Bad	67 (26.6)	9 (10.2)	58 (27.4)	
Relationship with mother ^b^				<0.001
Good	222 (88.1)	86 (97.7)	136 (82.9)	
Fair or Bad	30 (11.9)	2 (2.3)	28 (17.1)	
Making decisions without interference by father ^b^				0.932
Always	70 (27.8)	26 (29.5)	44 (26.8)	
Often	109 (43.3)	37 (42)	72 (43.9)	
Sometimes or occasionally	73 (29)	25 (28.4)	48 (29.3)	
Making decisions without interference by mother ^b^				0.655
Always	52 (20.6)	22 (25)	30 (18.3)	
Often	99 (39.3)	32 (36.4)	67 (40.9)	
Sometimes or occasionally	101 (40.1)	34 (38.6)	67 (40.9)	

^a^ Continuous predictors were calculated as the mean ± SD. ^b^ Categorical predictors were calculated as n (%).

**Table 2:** Proportion of depression levels among nursing students (N = 252).

Variable	Category	n	%
Depression Level	No to minimal depression	88	34.9
	Moderate depression	60	23.8
	Severe depression	104	41.3

**Table 3:** Odd ratios (95%CI) of depression for socio-demographic characteristics, education, and family-related factors in logistic regression models.

	Univariate Model ^a^		Multi-Variate Model ^b^	
	OR (95%CI)	*p*	OR (95%CI)	*p*
Sex		<0.001		
Male	0.29 (0.16–0.51)		0.42 (0.23–0.78)	0.006
Female	1		1	
Academic pressure				
Greater	5.87 (2.04–16.84)	<0.001	3.94 (1.23–12.54)	0.020
Medium	2.06 (0.75–5.68)	0.160	1.77 (0.58–5.38)	0.314
Less	1		1	
Interpersonal relationship		<0.001		<0.001
Good	0.32 (0.18–0.56)		0.36 (0.20–0.67)	
Fair or poor	1		1	
Relationship with father		<0.001		<0.001
Good	0.20 (0.09–0.44)		0.25 (0.11–0.58)	
Fair or bad	1		1	

^a.^ The univariate model involved a single factor. ^b.^ The four factors in this table were all included in the multi-variate framework by applying the backward method according to *p*_out_ b 0.10.

## Data Availability

Upon request to the corresponding author.

## Supplementary Materials

The following are available online at https://www.mdpi.com/article/10.3390/healthcare12131316/s1, Supplementary Table S1: Linear regression for exploring the association of depression.
