# Supplementary material for: Prevalence and Associations of Depression among Saudi College Nursing Students: A Cross-Sectional Study
Source: Healthcare (Basel). 2024 Jul 1;12(13):1316. doi: 10.3390/healthcare12131316 (PMC11240896; doi:10.3390/healthcare12131316)
Supplement: Supplementary file 1 [file healthcare-12-01316-s001.zip › healthcare-2936042-supplementary.pdf]

Supplementary Table 1. Linear regression for exploring the associations of depression.

Variables	B	SE	β	t	p	95% confidence interval	
						Lower limit	Upper limit
**Personal information**							
Constant	52.164	7.560		6.900	<0.001	37.270	67.058
Sex	-1.862	1.473	-0.075	-1.264	0.207	-4.763	1.040
Single child	0.557	4.755	0.006	0.117	0.907	-8.810	9.924
Place of residence before college	-1.075	2.694	-0.022	-0.399	0.690	-6.381	4.232
Grades	-0.804	2.242	-0.020	-0.359	-0.720	-5.222	3.614
Academic pressure	4.299	1.166	0.212	3.686	<0.001	2.001	6.597
Interest in nursing major	0.043	1.108	0.002	0.039	0.969	-2.140	2.227
Perspective on future career prospects	-0.197	1.662	-0.007	-0.118	0.906	-3.470	3.077
Interpersonal relationships	-4.753	1.446	-0.190	-3.287	<0.001	-7.603	-1.904
Exercise frequency	-1.459	0.751	-0.110	-1.940	0.053	-2.938	0.020
**Family background**							
Educational level of father	0.957	1.584	0.036	0.604	0.546	-2.164	4.078
Educational level of mother	-0.052	1.475	-0.002	-0.035	0.972	-2.957	2.853
Relationship with father	-5.292	1.763	-0.188	-3.003	0.003	-8.765	-1.820
Relationship with mother	-9.130	2.395	-0.238	-3.812	<0.001	-13.848	-4.412
Making decisions without interference by father	0.085	1.162	0.005	0.074	0.941	-2.203	2.374
Making decisions without interference by mother	1.050	1.186	0.064	0.885	0.377	-1.287	3.387

Note. R = .589, R^2 = .347, Adjusted R2 = .305, F (1, 236) = 8.349, p < .001.
